# (η^6^-Benzene)­dichlorido­[di­cyclo­hex­yl(4-iso­propylphen­yl)phosphane-κ*P*]ruthenium(II)

**DOI:** 10.1107/S2414314626008710

**Published:** 2026-09-03

**Authors:** Monsuru T. Kelani, Eric C. Hosten, Alfred Muller, Koop Lammertsma

**Affiliations:** a Department of Chemical Sciences, University of Johannesburg, Auckland Park, Johannesburg 2006, South Africa; b Department of Chemistry, Nelson Mandela University, Summerstrand, Port Elizabeth 6001, South Africa; Vienna University of Technology, Austria

**Keywords:** crystal structure, triclinic, ruthenium.

## Abstract

The title complex, [RuCl_2_(C_6_H_6_)(C_21_H_33_P)] or (η^6^-C_6_H_6_)((C_6_H_11_)_2_(^*i*^PrC_6_H_4_)P)RuCl_2_ crystallizes in the space group *P*1 with one mol­ecule in the asymmetric unit. The Ru^II^ atom is located at distances of 2.3830 (7), 2.3925 (8), 2.4269 (8), and 1.6919 (3) Å from the P, the two Cl ligands, and the centroid of the benzene mol­ecule, respectively. A cone angle of 159° was calculated for the steric pocket of the phosphane ligand. Positional disorder over two sets of sites in the methine and one of the methyl groups of the isopropyl substitutent resulted in a refined 0.528 (5):0.472 (5) ratio. The crystal packing is consolidated by non-classical C—H⋯Cl inter­actions.

## Abstract 2

The title complex exhibits a half-sandwich structure with a tripodal piano stool-like arrangement consolidated by non-classical intra- and inter­molecular inter­actions.

## Graphical abstract

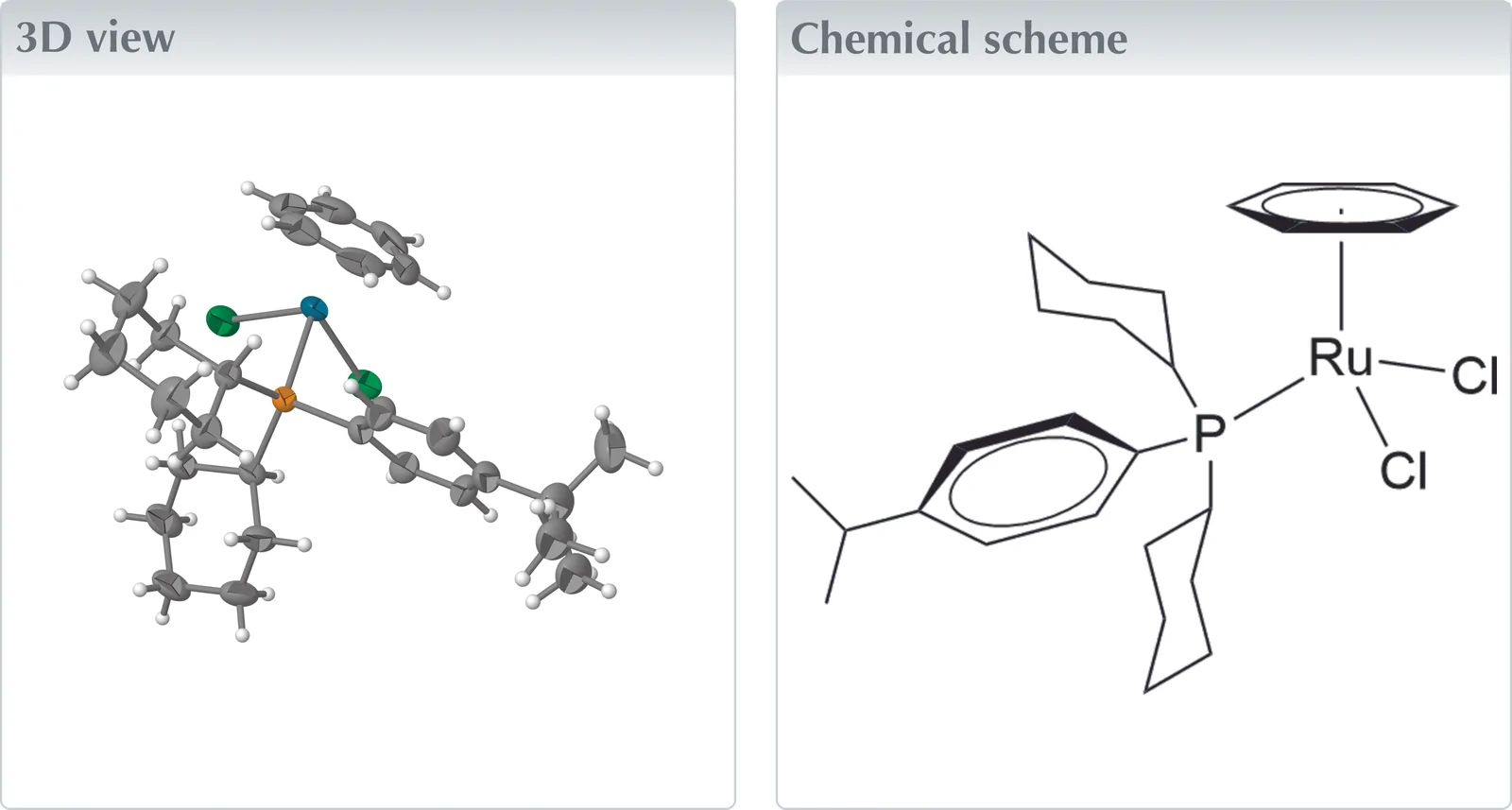

## Structure description

Arene-ruthenium complexes are known to demonstrate promising catalytic properties over a wide range of organic reactions (Faller & D’Alliessi, 2003 ▸). Moreover, if chirality is associated with arene-ruthenium complexes, they offer applications in asymmetric organic synthesis (Faller & D’Alliessi, 2003 ▸; Noyori *et al.*, 2001 ▸; Petra *et al.*, 2000 ▸; Haack *et al.*, 1997 ▸). In this context, we synthesized the title complex and determined its crystal structure.

The coordination of the central Ru^II^ atom shows the typical tripodal piano stool arrangement featuring two chlorido ligands, a di­cyclo­hex­yl(4-iso­propyl­phen­yl)phosphane ligand and a benzene mol­ecule, which completes the remaining coordination site (Fig. 1 ▸). The bond lengths [Ru—P = 2.3830 (7), Ru—Cl1 = 2.3925 (8), Ru—Cl2 = 2.4269 (8), Ru—benzene­(centroid) = 1.6919 (3) Å] and angles [Cl1—Ru1—Cl2 = 87.13 (3), Cl1—Ru1—P1 = 85.81 (3), Cl2—Ru1—P1 = 92.96 (3)°] are within reported values of closely related structural analogues, such as Ru(*η*^6^-C_6_H_6_)Cl_2_{P(C_6_H_11_)_2_*R*}, where *R* is a substituted phenyl ring (Makarova *et al.*, 2018 ▸; Muller & Davis, 2012 ▸; Granville *et al.*, 2012 ▸). The substituents of the phosphane ligands are arranged on the opposite side in a staggered pattern relative to the chlorido ligands to minimize steric inter­actions. The steric impact due to the phosphane ligand was qu­anti­fied as 159° for the effective Tolman cone angle (Müller & Mingos, 1995 ▸), which is similar to its analogue (158°; Muller & Davis, 2012 ▸) but smaller than its rhodium analogue with a value of 164°, where the *M*—P bond length was adjusted to 2.28 Å (Davis & Meijboom, 2011 ▸). Thus, owing to the steric demand of the phosphane ligand, the title complex might exhibit potential catalytic properties. The *η*^6^-bonding benzene mol­ecule is slightly skewed towards the phosphane ligand, a feature primarily observed for this system but not for the analogous *η*^6^-cymene system (do Rosario *et al.*, 2023 ▸). Non-classical intra- and inter­molecular C—H⋯Cl inter­actions (Table 1 ▸) consolidate the mol­ecular conformation. The Cl1 atom is an acceptor for two inter­molecular inter­actions C11—H11⋯Cl1 and C15—H15*B*⋯Cl1 linking mol­ecules in chains with *C*(7) and *C*(10) descriptors extending parallel to the *a* axis (Fig. 2 ▸). The crystal packing also exhibits parallel stacking between the ligating benzene rings with a large offset (Malenov & Zaric, 2020 ▸), here with a slippage of 4.49 Å. The separation between C2 and C2[2 − *x*, −*y*, 1 − *z*] is 3.172 (6) Å, 0.23 Å less that the sum of the van der Waals radii.

## Synthesis and crystallization

Di­cyclo­hex­yl(4-iso­propyl­phen­yl)phosphane (64 mg, 0.2 mmol, 2 eq) was added to a solution of [*η*^6^-C_6_H_6_)_2_RuCl_2_]_2_ (50 mg, 0.1 mmol, 1 eq) in methanol (2 ml) at room temperature under continuous stirring for 24 h. Yellow crystals of the title compound were obtained by slow evaporation of the reaction mixture upon cooling to ambient temperature.

## Refinement

Crystal data, data collection and structure refinement details are summarized in Table 2 ▸. The elongated displacement ellipsoids of C13 and C14 in the initial model were treated by a positional disorder model over two sets of sites, which refined to a 0.528 (5):0.472 (5) ratio for parts *A* and *B*. The shapes of the displacement ellipsoids and pseudo-flat feature of the *η*^6^-coordinating benzene mol­ecule indicates disorder, which could not be resolved on basis of the present data.

## Supplementary Material

Crystal structure: contains datablock(s) I. DOI: 10.1107/S2414314626008710/wm4255sup1.cif

Structure factors: contains datablock(s) I. DOI: 10.1107/S2414314626008710/wm4255Isup2.hkl

CCDC reference: 2582586

Additional supporting information:  crystallographic information; 3D view; checkCIF report

## Figures and Tables

**Figure 1:**
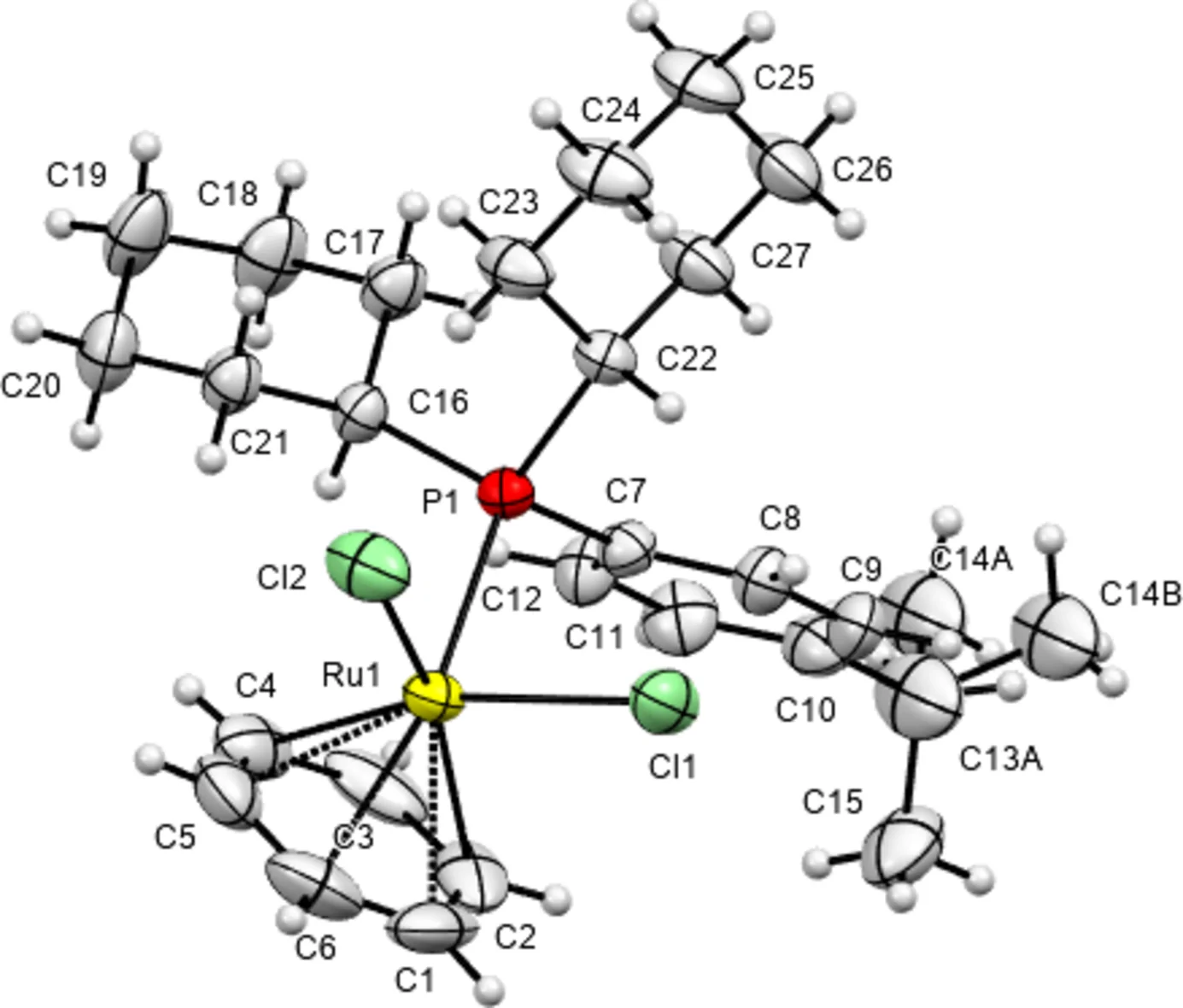
The mol­ecular structure of the title complex drawn with displacement ellipsoids at the 50% probability level. The two components of disorder of the isopropyl group are shown.

**Figure 2:**
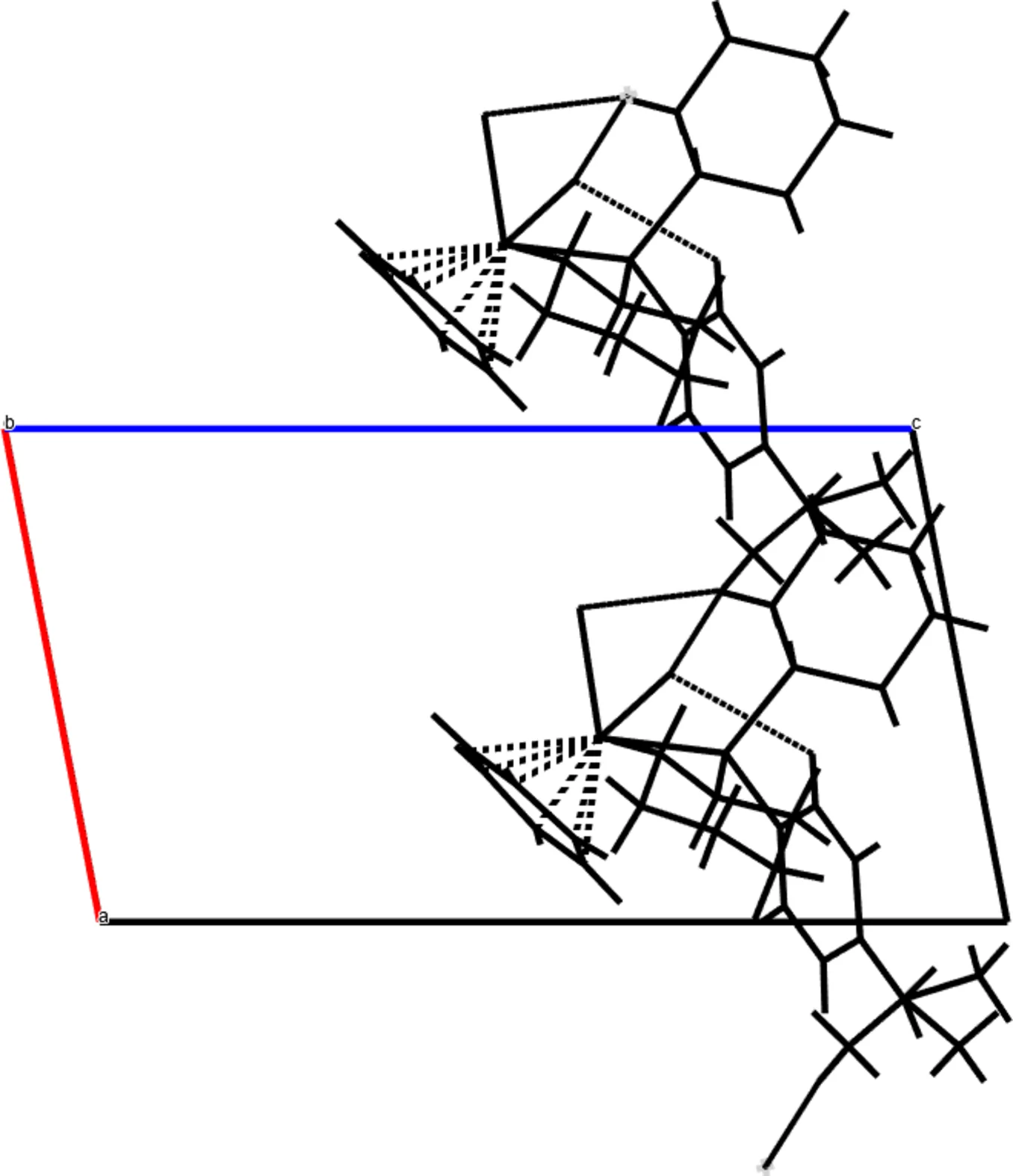
The packing of mol­ecules in a wireframe representation with the non-classical C—H⋯Cl inter­actions shown as dashed lines.

**Table 1:** Hydrogen-bond geometry (Å, °)

*D*—H⋯*A*	*D*—H	H⋯*A*	*D*⋯*A*	*D*—H⋯*A*
C11—H11⋯Cl1^i^	0.93	2.83	3.640 (3)	146
C8—H8⋯Cl1	0.93	2.79	3.388 (3)	123
C22—H22⋯Cl1	0.98	2.88	3.548 (3)	126
C23—H23*A*⋯Cl2	0.97	2.58	3.470 (3)	152
C15—H15*B*⋯Cl1^i^	0.96	2.72	3.656 (4)	164

Symmetry code: (i) 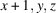.

**Table 2:** Experimental details

Crystal data	
Chemical formula	[RuCl_2_(C_6_H_6_)(C_21_H_33_P)]
*M*_r_	566.52
Crystal system, space group	Triclinic, *P* 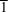
Temperature (K)	273
*a*, *b*, *c* (Å)	8.5992 (9), 10.2612 (12), 15.4390 (16)
α, β, γ (°)	84.711 (4), 78.450 (4), 81.738 (4)
*V* (Å^3^)	1318.0 (2)
*Z*	2
Radiation type	Mo *K*α
μ (mm^−1^)	0.87
Crystal size (mm)	0.39 × 0.25 × 0.09

Data collection	
Diffractometer	Bruker APEXII CCD
Absorption correction	Multi-scan (*SADABS*; Krause *et al.*, 2015 ▸)
*T*_min_, *T*_max_	0.690, 0.746
No. of measured, independent and observed [*I* > 2σ(*I*)] reflections	48604, 6556, 5292
*R*_int_	0.077
(sin θ/λ)_max_ (Å^−1^)	0.668

Refinement	
*R*[*F*^2^ > 2σ(*F*^2^)], *wR*(*F*^2^), *S*	0.037, 0.085, 1.03
No. of reflections	6556
No. of parameters	280
No. of restraints	13
H-atom treatment	H-atom parameters constrained
Δρ_max_, Δρ_min_ (e Å^−3^)	0.50, −0.47

Computer programs: *APEX2* and *SAINT* (Bruker, 2010 ▸), *SHELXT* (Sheldrick, 2015*a* ▸), *SHELXL* (Sheldrick, 2015*b* ▸), *Mercury* (Macrae *et al.*., 2020 ▸), *publCIF* (Westrip, 2010 ▸) and *WinGX* (Farrugia, 2012 ▸).

## full crystallographic data

### (η6-Benzene)dichlorido[dicyclohexyl(4-isopropylphenyl)phosphane-κP]ruthenium(II) Crystal data

**Table:**

[RuCl_2_(C_6_H_6_)(C_21_H_33_P)]	*Z* = 2
*M**_r_* = 566.52	*F*(000) = 588
Triclinic, *P*1	*D*_x_ = 1.427 Mg m^−^^3^
*a* = 8.5992 (9) Å	Mo *K*α radiation, λ = 0.71073 Å
*b* = 10.2612 (12) Å	Cell parameters from 6769 reflections
*c* = 15.4390 (16) Å	θ = 2.4–25.0°
α = 84.711 (4)°	µ = 0.87 mm^−^^1^
β = 78.450 (4)°	*T* = 273 K
γ = 81.738 (4)°	Block, yellow
*V* = 1318.0 (2) Å^3^	0.39 × 0.25 × 0.09 mm

### (η6-Benzene)dichlorido[dicyclohexyl(4-isopropylphenyl)phosphane-κP]ruthenium(II) Data collection

**Table:**

Bruker APEXII CCD diffractometer	6556 independent reflections
Radiation source: sealed tube	5292 reflections with *I* > 2σ(*I*)
Detector resolution: 8.3333 pixels mm^-1^	*R*_int_ = 0.077
φ and ω scans	θ_max_ = 28.3°, θ_min_ = 2.0°
Absorption correction: multi-scan (SADABS; Krause *et al.*, 2015)	*h* = −11→11
*T*_min_ = 0.690, *T*_max_ = 0.746	*k* = −13→13
48604 measured reflections	*l* = −20→20

### (η6-Benzene)dichlorido[dicyclohexyl(4-isopropylphenyl)phosphane-κP]ruthenium(II) Refinement

**Table:**

Refinement on *F*^2^	13 restraints
Least-squares matrix: full	Hydrogen site location: inferred from neighbouring sites
*R*[*F*^2^ > 2σ(*F*^2^)] = 0.037	H-atom parameters constrained
*wR*(*F*^2^) = 0.085	*w* = 1/[σ^2^(*F*_o_^2^) + (0.0311*P*)^2^ + 0.6372*P*] where *P* = (*F*_o_^2^ + 2*F*_c_^2^)/3
*S* = 1.03	(Δ/σ)_max_ = 0.001
6556 reflections	Δρ_max_ = 0.50 e Å^−^^3^
280 parameters	Δρ_min_ = −0.47 e Å^−^^3^

### (η6-Benzene)dichlorido[dicyclohexyl(4-isopropylphenyl)phosphane-κP]ruthenium(II) Special details

**Table:**

Geometry. All esds (except the esd in the dihedral angle between two l.s. planes) are estimated using the full covariance matrix. The cell esds are taken into account individually in the estimation of esds in distances, angles and torsion angles; correlations between esds in cell parameters are only used when they are defined by crystal symmetry. An approximate (isotropic) treatment of cell esds is used for estimating esds involving l.s. planes.

### (η6-Benzene)dichlorido[dicyclohexyl(4-isopropylphenyl)phosphane-κP]ruthenium(II) Fractional atomic coordinates and isotropic or equivalent isotropic displacement parameters (Å^2^)

**Table:**

	*x*	*y*	*z*	*U*_iso_*/*U*_eq_	Occ. (<1)
Ru1	0.62705 (2)	0.21226 (2)	0.58991 (2)	0.03363 (7)	
Cl1	0.49749 (9)	0.04255 (7)	0.68079 (5)	0.04836 (18)	
Cl2	0.36313 (9)	0.33631 (9)	0.59551 (5)	0.0565 (2)	
P1	0.65764 (7)	0.29085 (6)	0.72526 (4)	0.02889 (14)	
C1	0.7180 (5)	0.0682 (4)	0.4865 (3)	0.0701 (11)	
H1	0.688704	−0.013230	0.479014	0.084*	
C2	0.8305 (5)	0.0751 (4)	0.5409 (2)	0.0733 (12)	
H2	0.869452	0.000247	0.572719	0.088*	
C3	0.8829 (4)	0.2003 (5)	0.5459 (2)	0.0678 (11)	
H3	0.961601	0.207879	0.577930	0.081*	
C4	0.8110 (4)	0.3123 (4)	0.5005 (2)	0.0609 (9)	
H4	0.843354	0.394648	0.501952	0.073*	
C5	0.6950 (4)	0.2993 (4)	0.4550 (2)	0.0610 (9)	
H5	0.642590	0.374287	0.429584	0.073*	
C6	0.6533 (5)	0.1773 (5)	0.4458 (2)	0.0685 (11)	
H6	0.578944	0.170638	0.410667	0.082*	
C7	0.8084 (3)	0.1725 (2)	0.76968 (17)	0.0343 (5)	
C8	0.7646 (3)	0.0545 (3)	0.81327 (19)	0.0412 (6)	
H8	0.658933	0.038096	0.820859	0.049*	
C9	0.8752 (4)	−0.0383 (3)	0.8453 (2)	0.0480 (7)	
H9	0.841995	−0.115494	0.875088	0.058*	
C10	1.0343 (4)	−0.0199 (3)	0.83440 (19)	0.0478 (7)	
C11	1.0787 (3)	0.0958 (3)	0.7890 (2)	0.0503 (7)	
H11	1.185326	0.110166	0.779557	0.060*	
C12	0.9684 (3)	0.1905 (3)	0.7574 (2)	0.0435 (7)	
H12	1.002092	0.267478	0.727525	0.052*	
C15	1.2520 (5)	−0.2051 (3)	0.7984 (3)	0.0787 (12)	
H15A	1.181694	−0.236951	0.766449	0.118*	
H15B	1.323229	−0.153130	0.758479	0.118*	
H15C	1.313200	−0.278676	0.824388	0.118*	
C16	0.7472 (3)	0.4473 (2)	0.70614 (17)	0.0337 (5)	
H16	0.851384	0.425306	0.667669	0.040*	
C17	0.7862 (4)	0.5040 (3)	0.78672 (19)	0.0435 (7)	
H17A	0.687906	0.539441	0.824564	0.052*	
H17B	0.841137	0.434619	0.820791	0.052*	
C18	0.8923 (4)	0.6133 (3)	0.7556 (2)	0.0549 (8)	
H18A	0.912557	0.651367	0.806814	0.066*	
H18B	0.994354	0.575408	0.722486	0.066*	
C19	0.8160 (5)	0.7212 (3)	0.6977 (2)	0.0628 (9)	
H19A	0.891572	0.783387	0.674529	0.075*	
H19B	0.722988	0.768606	0.733399	0.075*	
C20	0.7655 (4)	0.6661 (3)	0.6210 (2)	0.0525 (8)	
H20A	0.860091	0.630718	0.580263	0.063*	
H20B	0.707874	0.736749	0.589202	0.063*	
C21	0.6591 (3)	0.5574 (3)	0.65316 (19)	0.0403 (6)	
H21A	0.632313	0.521791	0.602769	0.048*	
H21B	0.560433	0.593842	0.690209	0.048*	
C22	0.4837 (3)	0.3030 (3)	0.81931 (16)	0.0335 (5)	
H22	0.430647	0.224682	0.819080	0.040*	
C23	0.3582 (3)	0.4216 (3)	0.80847 (19)	0.0482 (7)	
H23A	0.328775	0.422976	0.750897	0.058*	
H23B	0.403024	0.502284	0.811225	0.058*	
C24	0.2091 (4)	0.4156 (4)	0.8809 (2)	0.0601 (9)	
H24A	0.133000	0.493402	0.873799	0.072*	
H24B	0.159170	0.338802	0.874793	0.072*	
C25	0.2494 (4)	0.4079 (4)	0.9729 (2)	0.0635 (10)	
H25A	0.287126	0.489383	0.981904	0.076*	
H25B	0.153957	0.397323	1.017157	0.076*	
C26	0.3768 (4)	0.2933 (4)	0.9835 (2)	0.0612 (9)	
H26A	0.333974	0.211419	0.981463	0.073*	
H26B	0.405832	0.293605	1.041066	0.073*	
C27	0.5256 (4)	0.2993 (3)	0.91169 (18)	0.0488 (7)	
H27A	0.602949	0.222623	0.919706	0.059*	
H27B	0.573841	0.377440	0.916662	0.059*	
C13A	1.1560 (5)	−0.1225 (4)	0.8695 (3)	0.0812 (11)	0.528 (5)
H13A	1.093279	−0.182559	0.910643	0.097*	0.528 (5)
C14A	1.2510 (9)	−0.0713 (8)	0.9202 (5)	0.0812 (11)	0.528 (5)
H14A	1.323818	−0.141731	0.940661	0.122*	0.528 (5)
H14B	1.183038	−0.030280	0.970102	0.122*	0.528 (5)
H14C	1.310592	−0.007079	0.884063	0.122*	0.528 (5)
C13B	1.1560 (5)	−0.1225 (4)	0.8695 (3)	0.0812 (11)	0.472 (5)
H13B	1.234608	−0.067664	0.878998	0.097*	0.472 (5)
C14B	1.1085 (10)	−0.1823 (8)	0.9574 (5)	0.0812 (11)	0.472 (5)
H14D	1.047237	−0.252506	0.954713	0.122*	0.472 (5)
H14E	1.044669	−0.117131	0.994811	0.122*	0.472 (5)
H14F	1.202176	−0.217108	0.981138	0.122*	0.472 (5)

### (η6-Benzene)dichlorido[dicyclohexyl(4-isopropylphenyl)phosphane-κP]ruthenium(II) Atomic displacement parameters (Å^2^)

**Table:**

	*U*^11^	*U*^22^	*U*^33^	*U*^12^	*U*^13^	*U*^23^
Ru1	0.03321 (12)	0.03697 (13)	0.02956 (11)	−0.00424 (8)	−0.00077 (8)	−0.00810 (8)
Cl1	0.0535 (4)	0.0469 (4)	0.0461 (4)	−0.0209 (3)	0.0000 (3)	−0.0086 (3)
Cl2	0.0472 (4)	0.0749 (6)	0.0452 (4)	0.0129 (4)	−0.0144 (3)	−0.0116 (4)
P1	0.0281 (3)	0.0287 (3)	0.0295 (3)	−0.0019 (2)	−0.0049 (2)	−0.0038 (2)
C1	0.078 (3)	0.057 (2)	0.067 (2)	−0.0185 (19)	0.027 (2)	−0.0365 (19)
C2	0.068 (2)	0.068 (2)	0.053 (2)	0.034 (2)	0.0277 (17)	0.0062 (18)
C3	0.0285 (15)	0.130 (4)	0.0412 (18)	−0.0073 (19)	0.0071 (13)	−0.024 (2)
C4	0.062 (2)	0.064 (2)	0.0518 (19)	−0.0275 (18)	0.0211 (16)	−0.0156 (17)
C5	0.065 (2)	0.073 (2)	0.0359 (16)	−0.0038 (18)	0.0062 (15)	0.0042 (16)
C6	0.067 (2)	0.103 (3)	0.0373 (18)	−0.014 (2)	0.0008 (16)	−0.027 (2)
C7	0.0339 (13)	0.0319 (13)	0.0357 (13)	0.0010 (10)	−0.0063 (11)	−0.0040 (11)
C8	0.0421 (15)	0.0375 (15)	0.0450 (16)	−0.0079 (12)	−0.0099 (12)	−0.0002 (12)
C9	0.062 (2)	0.0344 (15)	0.0443 (16)	0.0023 (13)	−0.0107 (14)	0.0031 (12)
C10	0.0479 (17)	0.0486 (18)	0.0407 (16)	0.0162 (14)	−0.0087 (13)	−0.0047 (13)
C11	0.0325 (15)	0.0561 (19)	0.0594 (19)	0.0033 (13)	−0.0096 (13)	−0.0010 (15)
C12	0.0352 (14)	0.0413 (16)	0.0514 (17)	−0.0028 (12)	−0.0068 (13)	0.0035 (13)
C15	0.072 (3)	0.048 (2)	0.102 (3)	0.0113 (18)	0.003 (2)	−0.002 (2)
C16	0.0358 (13)	0.0295 (13)	0.0366 (13)	−0.0055 (10)	−0.0080 (11)	−0.0015 (10)
C17	0.0509 (17)	0.0362 (15)	0.0478 (17)	−0.0057 (12)	−0.0195 (13)	−0.0032 (12)
C18	0.062 (2)	0.0444 (17)	0.070 (2)	−0.0196 (15)	−0.0326 (17)	0.0013 (15)
C19	0.080 (2)	0.0367 (17)	0.081 (3)	−0.0219 (16)	−0.031 (2)	0.0052 (16)
C20	0.062 (2)	0.0409 (17)	0.0575 (19)	−0.0132 (14)	−0.0211 (16)	0.0118 (14)
C21	0.0456 (16)	0.0358 (14)	0.0413 (15)	−0.0043 (12)	−0.0153 (12)	0.0018 (12)
C22	0.0320 (13)	0.0375 (14)	0.0298 (12)	−0.0030 (10)	−0.0023 (10)	−0.0056 (10)
C23	0.0441 (16)	0.0598 (19)	0.0363 (15)	0.0120 (14)	−0.0080 (12)	−0.0086 (13)
C24	0.0373 (16)	0.086 (3)	0.0535 (19)	0.0088 (16)	−0.0037 (14)	−0.0210 (18)
C25	0.0491 (19)	0.087 (3)	0.0459 (18)	0.0057 (18)	0.0071 (15)	−0.0200 (18)
C26	0.066 (2)	0.079 (2)	0.0313 (15)	−0.0003 (18)	0.0000 (15)	−0.0044 (15)
C27	0.0460 (17)	0.067 (2)	0.0299 (14)	0.0061 (14)	−0.0076 (12)	−0.0056 (13)
C13A	0.085 (2)	0.086 (2)	0.064 (2)	0.0366 (19)	−0.0274 (17)	0.0002 (17)
C14A	0.085 (2)	0.086 (2)	0.064 (2)	0.0366 (19)	−0.0274 (17)	0.0002 (17)
C13B	0.085 (2)	0.086 (2)	0.064 (2)	0.0366 (19)	−0.0274 (17)	0.0002 (17)
C14B	0.085 (2)	0.086 (2)	0.064 (2)	0.0366 (19)	−0.0274 (17)	0.0002 (17)

### (η6-Benzene)dichlorido[dicyclohexyl(4-isopropylphenyl)phosphane-κP]ruthenium(II) Geometric parameters (Å, º)

**Table:**

Ru1—P1	2.3830 (7)	C17—H17B	0.9700
Ru1—Cl1	2.3925 (8)	C18—C19	1.516 (4)
Ru1—Cl2	2.4269 (8)	C18—H18A	0.9700
P1—C7	1.837 (3)	C18—H18B	0.9700
P1—C16	1.853 (2)	C19—C20	1.520 (4)
P1—C22	1.863 (2)	C19—H19A	0.9700
C1—C6	1.338 (5)	C19—H19B	0.9700
C1—C2	1.415 (6)	C20—C21	1.528 (4)
C1—H1	0.9300	C20—H20A	0.9700
C2—C3	1.435 (5)	C20—H20B	0.9700
C2—H2	0.9300	C21—H21A	0.9700
C3—C4	1.420 (5)	C21—H21B	0.9700
C3—H3	0.9300	C22—C23	1.527 (4)
C4—C5	1.357 (5)	C22—C27	1.535 (4)
C4—H4	0.9300	C22—H22	0.9800
C5—C6	1.379 (5)	C23—C24	1.528 (4)
C5—H5	0.9300	C23—H23A	0.9700
C6—H6	0.9300	C23—H23B	0.9700
C7—C12	1.388 (4)	C24—C25	1.520 (4)
C7—C8	1.393 (4)	C24—H24A	0.9700
C8—C9	1.378 (4)	C24—H24B	0.9700
C8—H8	0.9300	C25—C26	1.509 (5)
C9—C10	1.383 (4)	C25—H25A	0.9700
C9—H9	0.9300	C25—H25B	0.9700
C10—C11	1.384 (4)	C26—C27	1.521 (4)
C10—C13B	1.517 (4)	C26—H26A	0.9700
C10—C13A	1.517 (4)	C26—H26B	0.9700
C11—C12	1.384 (4)	C27—H27A	0.9700
C11—H11	0.9300	C27—H27B	0.9700
C12—H12	0.9300	C13A—C14A	1.419 (7)
C15—C13B	1.487 (5)	C13A—H13A	0.9800
C15—C13A	1.487 (5)	C14A—H14A	0.9600
C15—H15A	0.9600	C14A—H14B	0.9600
C15—H15B	0.9600	C14A—H14C	0.9600
C15—H15C	0.9600	C13B—C14B	1.443 (8)
C16—C21	1.533 (3)	C13B—H13B	0.9800
C16—C17	1.535 (4)	C14B—H14D	0.9600
C16—H16	0.9800	C14B—H14E	0.9600
C17—C18	1.529 (4)	C14B—H14F	0.9600
C17—H17A	0.9700		
			
P1—Ru1—Cl1	85.81 (3)	C20—C19—H19B	109.2
P1—Ru1—Cl2	92.95 (3)	H19A—C19—H19B	107.9
Cl1—Ru1—Cl2	87.12 (3)	C19—C20—C21	111.5 (3)
C7—P1—C16	104.05 (12)	C19—C20—H20A	109.3
C7—P1—C22	103.40 (12)	C21—C20—H20A	109.3
C16—P1—C22	110.12 (12)	C19—C20—H20B	109.3
C7—P1—Ru1	107.20 (8)	C21—C20—H20B	109.3
C16—P1—Ru1	111.58 (8)	H20A—C20—H20B	108.0
C22—P1—Ru1	119.04 (8)	C20—C21—C16	109.7 (2)
C6—C1—C2	120.9 (3)	C20—C21—H21A	109.7
C6—C1—H1	119.6	C16—C21—H21A	109.7
C2—C1—H1	119.6	C20—C21—H21B	109.7
C1—C2—C3	118.4 (3)	C16—C21—H21B	109.7
C1—C2—H2	120.8	H21A—C21—H21B	108.2
C3—C2—H2	120.8	C23—C22—C27	109.0 (2)
C4—C3—C2	118.0 (3)	C23—C22—P1	113.61 (19)
C4—C3—H3	121.0	C27—C22—P1	115.48 (18)
C2—C3—H3	121.0	C23—C22—H22	106.0
C5—C4—C3	120.0 (3)	C27—C22—H22	106.0
C5—C4—H4	120.0	P1—C22—H22	106.0
C3—C4—H4	120.0	C22—C23—C24	111.0 (3)
C4—C5—C6	121.4 (4)	C22—C23—H23A	109.4
C4—C5—H5	119.3	C24—C23—H23A	109.4
C6—C5—H5	119.3	C22—C23—H23B	109.4
C1—C6—C5	121.0 (4)	C24—C23—H23B	109.4
C1—C6—H6	119.5	H23A—C23—H23B	108.0
C5—C6—H6	119.5	C25—C24—C23	111.6 (3)
C12—C7—C8	117.3 (2)	C25—C24—H24A	109.3
C12—C7—P1	123.2 (2)	C23—C24—H24A	109.3
C8—C7—P1	119.4 (2)	C25—C24—H24B	109.3
C9—C8—C7	121.1 (3)	C23—C24—H24B	109.3
C9—C8—H8	119.5	H24A—C24—H24B	108.0
C7—C8—H8	119.5	C26—C25—C24	110.5 (3)
C8—C9—C10	121.8 (3)	C26—C25—H25A	109.6
C8—C9—H9	119.1	C24—C25—H25A	109.6
C10—C9—H9	119.1	C26—C25—H25B	109.6
C9—C10—C11	117.2 (3)	C24—C25—H25B	109.6
C9—C10—C13B	121.7 (3)	H25A—C25—H25B	108.1
C11—C10—C13B	121.2 (3)	C25—C26—C27	112.0 (3)
C9—C10—C13A	121.7 (3)	C25—C26—H26A	109.2
C11—C10—C13A	121.2 (3)	C27—C26—H26A	109.2
C10—C11—C12	121.7 (3)	C25—C26—H26B	109.2
C10—C11—H11	119.2	C27—C26—H26B	109.2
C12—C11—H11	119.2	H26A—C26—H26B	107.9
C11—C12—C7	121.0 (3)	C26—C27—C22	110.8 (2)
C11—C12—H12	119.5	C26—C27—H27A	109.5
C7—C12—H12	119.5	C22—C27—H27A	109.5
C13A—C15—H15A	109.5	C26—C27—H27B	109.5
C13A—C15—H15B	109.5	C22—C27—H27B	109.5
H15A—C15—H15B	109.5	H27A—C27—H27B	108.1
C13A—C15—H15C	109.5	C14A—C13A—C15	113.2 (5)
H15A—C15—H15C	109.5	C14A—C13A—C10	114.2 (4)
H15B—C15—H15C	109.5	C15—C13A—C10	111.7 (3)
C21—C16—C17	109.4 (2)	C14A—C13A—H13A	105.6
C21—C16—P1	114.77 (17)	C15—C13A—H13A	105.6
C17—C16—P1	117.37 (18)	C10—C13A—H13A	105.6
C21—C16—H16	104.6	C13A—C14A—H14A	109.5
C17—C16—H16	104.6	C13A—C14A—H14B	109.5
P1—C16—H16	104.6	H14A—C14A—H14B	109.5
C18—C17—C16	109.6 (2)	C13A—C14A—H14C	109.5
C18—C17—H17A	109.7	H14A—C14A—H14C	109.5
C16—C17—H17A	109.7	H14B—C14A—H14C	109.5
C18—C17—H17B	109.7	C14B—C13B—C15	119.6 (5)
C16—C17—H17B	109.7	C14B—C13B—C10	117.2 (4)
H17A—C17—H17B	108.2	C15—C13B—C10	111.7 (3)
C19—C18—C17	112.1 (3)	C14B—C13B—H13B	101.4
C19—C18—H18A	109.2	C15—C13B—H13B	101.4
C17—C18—H18A	109.2	C10—C13B—H13B	101.4
C19—C18—H18B	109.2	C13B—C14B—H14D	109.5
C17—C18—H18B	109.2	C13B—C14B—H14E	109.5
H18A—C18—H18B	107.9	H14D—C14B—H14E	109.5
C18—C19—C20	111.9 (3)	C13B—C14B—H14F	109.5
C18—C19—H19A	109.2	H14D—C14B—H14F	109.5
C20—C19—H19A	109.2	H14E—C14B—H14F	109.5
C18—C19—H19B	109.2		
			
C6—C1—C2—C3	−4.8 (5)	C21—C16—C17—C18	−59.9 (3)
C1—C2—C3—C4	4.1 (4)	P1—C16—C17—C18	167.1 (2)
C2—C3—C4—C5	0.8 (4)	C16—C17—C18—C19	56.3 (4)
C3—C4—C5—C6	−5.2 (5)	C17—C18—C19—C20	−53.1 (4)
C2—C1—C6—C5	0.5 (5)	C18—C19—C20—C21	53.3 (4)
C4—C5—C6—C1	4.7 (5)	C19—C20—C21—C16	−57.1 (3)
C16—P1—C7—C12	−21.0 (3)	C17—C16—C21—C20	60.5 (3)
C22—P1—C7—C12	−136.1 (2)	P1—C16—C21—C20	−165.2 (2)
Ru1—P1—C7—C12	97.3 (2)	C7—P1—C22—C23	164.8 (2)
C16—P1—C7—C8	163.1 (2)	C16—P1—C22—C23	54.1 (2)
C22—P1—C7—C8	48.0 (2)	Ru1—P1—C22—C23	−76.6 (2)
Ru1—P1—C7—C8	−78.6 (2)	C7—P1—C22—C27	37.7 (2)
C12—C7—C8—C9	2.1 (4)	C16—P1—C22—C27	−73.0 (2)
P1—C7—C8—C9	178.2 (2)	Ru1—P1—C22—C27	156.37 (18)
C7—C8—C9—C10	−1.2 (5)	C27—C22—C23—C24	−57.4 (3)
C8—C9—C10—C11	−0.5 (4)	P1—C22—C23—C24	172.2 (2)
C8—C9—C10—C13B	−179.8 (3)	C22—C23—C24—C25	57.0 (4)
C8—C9—C10—C13A	−179.8 (3)	C23—C24—C25—C26	−54.8 (4)
C9—C10—C11—C12	1.3 (5)	C24—C25—C26—C27	55.2 (4)
C13B—C10—C11—C12	−179.5 (3)	C25—C26—C27—C22	−57.3 (4)
C13A—C10—C11—C12	−179.5 (3)	C23—C22—C27—C26	57.4 (3)
C10—C11—C12—C7	−0.4 (5)	P1—C22—C27—C26	−173.2 (2)
C8—C7—C12—C11	−1.3 (4)	C9—C10—C13A—C14A	−130.1 (5)
P1—C7—C12—C11	−177.2 (2)	C11—C10—C13A—C14A	50.7 (6)
C7—P1—C16—C21	169.38 (19)	C9—C10—C13A—C15	99.8 (4)
C22—P1—C16—C21	−80.4 (2)	C11—C10—C13A—C15	−79.4 (5)
Ru1—P1—C16—C21	54.1 (2)	C9—C10—C13B—C14B	−43.6 (7)
C7—P1—C16—C17	−60.1 (2)	C11—C10—C13B—C14B	137.3 (6)
C22—P1—C16—C17	50.2 (2)	C9—C10—C13B—C15	99.8 (4)
Ru1—P1—C16—C17	−175.32 (18)	C11—C10—C13B—C15	−79.4 (5)

### (η6-Benzene)dichlorido[dicyclohexyl(4-isopropylphenyl)phosphane-κP]ruthenium(II) Hydrogen-bond geometry (Å, º)

**Table:**

*D*—H···*A*	*D*—H	H···*A*	*D*···*A*	*D*—H···*A*
C11—H11···Cl1^i^	0.93	2.83	3.640 (3)	146
C8—H8···Cl1	0.93	2.79	3.388 (3)	123
C22—H22···Cl1	0.98	2.88	3.548 (3)	126
C23—H23*A*···Cl2	0.97	2.58	3.470 (3)	152
C15—H15*B*···Cl1^i^	0.96	2.72	3.656 (4)	164

Symmetry code: (i) *x*+1, *y*, *z*.
